# Stillbirths and neonatal mortality in LMICs: A community-based mother-infant cohort study

**DOI:** 10.7189/jogh.13.04031

**Published:** 2023-04-14

**Authors:** Lison Rambliere, Agathe de Lauzanne, Jean-Baptiste Diouf, Andrianirina Zafitsara Zo, Myriam Landau, Perlinot Herindrainy, Delphine Hivernaud, Fatoumata Diene Sarr, Touch Sok, Muriel Vray, Jean-Marc Collard, Laurence Borand, Elisabeth Delarocque-Astagneau, Didier Guillemot, Elsa Kermorvant-Duchemin, Bich-Tram Huynh, BIRDY study group, Aina Nirina Randriamamonjiarison, Tanjona Antsa Volahasina, Fanjalalaina Rasoanaivo, Feno Manitra Jacob Rakotoarimanana, Tanjona Bodonirina Raheliarivao, Frédérique Randrianirina, Andriniaina Rakotondrasoa, Thida Chon, Sophie Goyet, Patrice Piola, Alexandra Kerleguer, Véronique Ngo, Siyin Lach, Pring Long, Arnaud Tarantola, Raymond Bercion, Amy Gassama Sow, Marguerite Diatta, Abibatou Ndiaye, Joseph Faye, Vincent Richard, Abdoulaye Seck, Pape Samba Dieye, Balla Sy, Bouya Ndao, Michael Padget, Armiya Youssouf Abdou, Benoit Garin

**Affiliations:** 1 Institut Pasteur, Université Paris Cité, Epidemiology and Modelling of Antibiotic Evasion (EMAE), Paris, France; 2 Université Paris-Saclay, UVSQ, Inserm, CESP, Anti-infective evasion and pharmacoepidemiology team, Montigny-le-Bretonneux, France; 3 Institut Pasteur du Cambodge, Epidemiology & Public Health Unit, Phnom Penh, Cambodia; 4 Centre Hospitalier Roi Baudouin Guédiawaye, Dakar, Senegal; 5 Peadiatric Ward, Centre Hospitalier de Soavinandriana, Antananarivo, Madagascar; 6 Institut Pasteur de Madagascar, Unité d’épidémiologie et de recherche clinique, Antananarivo, Madagascar; 7 Assistance Publique–Hôpitaux de Paris, Hôpital Universitaire Necker-Enfants Malades, Department of Neonatology, Université de Paris, Paris, France; 8 Institut Pasteur de Dakar, Unité d’épidémiologie des maladies infectieuses, Dakar, Senegal; 9 Ministry of Health, Phnom Penh, Cambodia; 10 Institut Pasteur de Madagascar, Unité de bactériologie expérimentale, Antananarivo, Madagascar; 11 Center for Tuberculosis Research, Division of Infectious Diseases, Johns Hopkins University School of Medicine, Baltimore, Maryland, USA; 12 APHP, GHU Université Paris-Saclay, Raymond Poincaré Hospital, Epidemiology and Public Health, Garches, France

## Abstract

**Background:**

The exact timing, causes, and circumstances of stillbirth and neonatal mortality in low- and middle-income countries (LMICs) remain poorly described, especially for antenatal stillbirths and deaths occurring at home. We aimed to provide reliable estimates of the incidence of stillbirth and neonatal death in three LMICs (Madagascar, Cambodia and Senegal) and to identify their main causes and associated risk factors.

**Methods:**

This study is based on data from an international, multicentric, prospective, longitudinal, community-based mother-infant cohort. We included pregnant mothers and prospectively followed up their children in the community. Stillbirths and deaths were systematically reported; information across healthcare settings was collected and verbal autopsies were performed to document the circumstances and timing of death.

**Results:**

Among the 4436 pregnancies and 4334 live births, the peripartum period and the first day of life were the key periods of mortality. The estimated incidence of stillbirth was 11 per 1000 total births in Cambodia, 15 per 1000 in Madagascar, and 12 per 1000 in Senegal. We estimated neonatal mortality at 18 per 1000 live births in Cambodia, 24 per 1000 in Madagascar, and 23 per 1000 in Senegal. Based on ultrasound biometric data, 16.1% of infants in Madagascar were born prematurely, where 42% of deliveries and 33% of deaths occurred outside healthcare facilities. Risk factors associated with neonatal death were mainly related to delivery or to events that newborns faced during the first week of life.

**Conclusions:**

These findings underscore the immediate need to improve care for and monitoring of children at birth and during early life to decrease infant mortality. Surveillance of stillbirth and neonatal mortality and their causes should be improved to mitigate this burden in LMICs.

Mortality of children under five years of age has declined by an estimated 3.6% over the past two decades, yet neonatal mortality (during 0-28 days following birth) and stillbirths (death before birth) have declined by only 2.5% and 1.8%, respectively [1]. The neonatal period alone accounts for an estimated 47% of deaths under five years of age [2]. The peripartum (labour and delivery) and postpartum periods are particularly critical, accounting for half of all stillbirths and more than one-third of neonatal deaths [1,2].

Most stillbirths and neonatal deaths occur in low- and middle-income countries (LMICs). While in 2019 there were an estimated three stillbirths per 1000 total births in high-income countries (HICs), this estimate was seven times higher in low-income countries (LICs) (21 per 1000), where the incidence of neonatal mortality was nine times higher (27 per 1000 live births) than in HICs (three per 1000) [3].

The World Health Organization (WHO) defines a stillborn baby as one born without signs of life after 28 weeks of gestation (WG) [4], but many countries use a cut-off of 22WG or rely on minimum birth weight thresholds when gestational age estimation is unreliable or unavailable [5]. The few published estimates available suggest that one-third of stillbirths follow complications at delivery and three-quarters are associated with maternal conditions (e.g., hypertension, antepartum haemorrhage, infection) [5-7].

Neonatal deaths are often better documented than stillbirths and include complications related to prematurity (35%) or difficult delivery (22%), and infections such as sepsis, meningitis, and pneumonia (22%) [8]. As approximately half of all deaths occur outside of health care facilities, they are often unrecognized and undocumented, and therefore not captured by surveillance systems [9]. Further, many LMICs lack adequate vital records, which remains the primary challenge for robust mortality estimation [6,8]. When deaths are registered, early neonatal death and stillbirths are generally combined as “perinatal mortality”, precluding separate estimates and identification of the specific causes and risk factors for each outcome [5]. To guide future public health interventions in LMICs, deaths both within and outside of health care facilities must be comprehensively documented to enable clear identification of possible causes of death and associated risk factors, and identification of incidence of stillbirth and neonatal mortality.

We aimed to estimate the incidence of stillbirths and neonatal deaths, causes, and risk factors, considering deaths occurring both in and outside of health facilities in three LMICs: Cambodia, Madagascar, and Senegal.

## METHODS

### Study design and data collection

We based this study on the data from the Bacterial Infections and Antibiotic Resistant Diseases in Young Children (BIRDY) cohort studies which aimed to determine the incidence of antibiotic-resistant infection in young infants across LMICs. We selected Senegal, Madagascar, and Cambodia as study countries because they are LMICs located in different world regions, each contains an Institut Pasteur with a microbiology laboratory and an epidemiology unit, facilitating the implementation of the study, and our funders were willing to fund the study in each of these countries.

Together, the BIRDY studies include data from BIRDY1 (Madagascar, Cambodia, and Senegal, 2012-2018) and BIRDY2 (Madagascar, 2018-2021). The study areas in Cambodia were the Steung Meanchey district of Phnom Penh (urban) and two districts of Kampong Speu province (rural), 50 km west of Phnom Penh. In Madagascar, the study area included three districts of the third arrondissement of Antananarivo (urban) and the district of Moramanga (rural), 115 km from Antananarivo. The study area in Senegal included the department of Guédiawaye in the Dakar region (urban) and the department of Foundiougne in the Fatick region (rural), 150 km from Dakar. This study is a standardised, population-based, prospective cohort study. We used standardised procedures and questionnaires across countries and sites and trained participating health care providers using identical training materials and provided them with identical health care resources (e.g., scales, growth charts). The protocols for BIRDY1 and BIRDY2 were identical, with the exception that pregnant women were recruited in the first trimester of pregnancy in BIRDY2 rather than in the third trimester of pregnancy in BIRDY1 (Supplementary Figure 1 in the **Online Supplementary Document**). We previously described the maternal inclusion criteria and the protocol for infant follow-up between birth and three months of age elsewhere [10,11]. Briefly, we exhaustively identified pregnant women in the geographic study areas through community health care workers. We included pregnant women monthly until delivery and newborns at birth whom we followed up by regular home visits (at birth and then at three, seven, 14, 28 and 60 days). We collected standardized data regarding socio-demographic data, clinical signs, anthropometric data, symptoms, and care pathways at each home visit. Among mothers attending partner health care facilities at delivery or in case of health events, medical staff documented standardized data, including delivery circumstances, symptoms, diagnosis severity, and treatment prescribed. When a death occurred in a health centre, we retrieved the medical record and the diagnosis, clinical data, and circumstances of death, as well as any additional observations. When a death occurred outside of a health facility, we conducted standardized verbal autopsies with the parent(s), following WHO recommendations [12]. Two to three independent paediatricians (double-blind) reviewed all cases to identify the suspected cause of death.

In the BIRDY2 cohort (Madagascar only), three prenatal visits (one per trimester, the first being the earliest possible) with a health care worker were performed, including three ultrasound screenings. A sonographer (DH) reviewed random sample of 10% of ultrasound images to assess image quality and reliability. Ultrasound scans allowed estimation of gestational age, assessment of foetal presentation, and detection of amniotic fluid volume abnormalities.

### Outcomes and definitions

**Figure 1:**
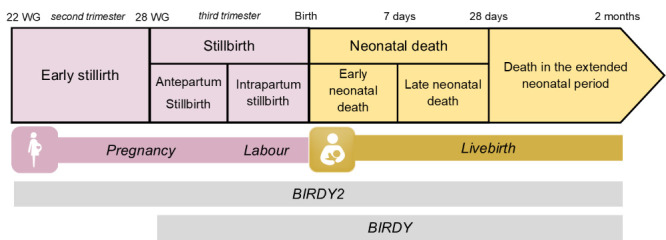
Definitions of stillbirth and deaths used in this study according to international recommendations; outcomes assessed were early stillbirth (22-28 WG, BIRDY2 only), stillbirth (>28 WG), early neonatal death (0-7 days), and late (8-28 days) and extended neonatal death (29 days to 2 months), as defined according to international definitions. Stillbirths were classified as antepartum if they occurred prior to labour and intrapartum if they occurred after labour onset. WG – weeks of gestation, d – days, m – months.

We presented the assessed outcomes in **Figure 1**. We collected details on stillbirth circumstances for Madagascar and Cambodia, but excluded the ones from as we determined they were unreliable. Details on neonatal death were available in all three countries. We considered birth before 37 completed WG as preterm.

### Statistical analyses

We presented the results as count (percentage) or mean (standard deviation) for normally distributed variables and median (interquartile range) for non-normally distributed variables. For descriptive statistics, we compared proportions using χ^2^ tests, means using Student’s tests, and medians using Kruskal-Wallis tests. We estimated the probability of survival over time with a Kaplan-Meier curve. We calculated incidences of stillbirth and neonatal mortality and their corresponding 95% confidence intervals (CIs).

We selected risk factors for stillbirth and neonatal death based on data from published literature. To maximize sample size, we pooled data across countries and forced the country variable in all models. We performed univariate logistic regression, subsequently including variables with *P* < 0.20 in a multivariate logistic regression model. We performed backwards stepwise selection for the final variable selection. The final measure of associations presented is the adjusted odds ratio (aOR) and its 95% CIs and *P*-value. We assessed risk factors for stillbirth (>28WG) and early neonatal death, but not for late and extended neonatal death due to an insufficient number of events. Prematurity status was not available in BIRDY1, so we used low birth weight (<2500g) as a proxy for prematurity in these analyses.

Overall, we determined that 1% of data were missing due to non-random reasons (i.e., due to greater omission among sex and birth weight variables in stillborn/dead babies). We handled this missing data using multiple imputations [13] for 63 cases following the procedure described by Von Hippel [14]. We estimated aORs across each of the imputed data sets and combined them using Rubin's rules [13,14].

### Role of the funding source

This study was supported by internal resources from Paris-Sud University. The BIRDY 1 & 2 research projects were implemented with the financial support of the Monegasque Cooperation for development, the Total Foundation, and MSDAVENIR.

### Ethics

The study was authorized by Institut Pasteur in Paris and by the Ethics Committees of each country (Madagascar (068-MSANP/CE and No119-MSANP/CERBM), Senegal (SEN 14-20), Cambodia (108 NEHCR) and the Institutional Review Board of Institut Pasteur (IRB/2016/08/03)). We obtained written informed consent from all participants.

## RESULTS

### Study population

**Figure 2:**
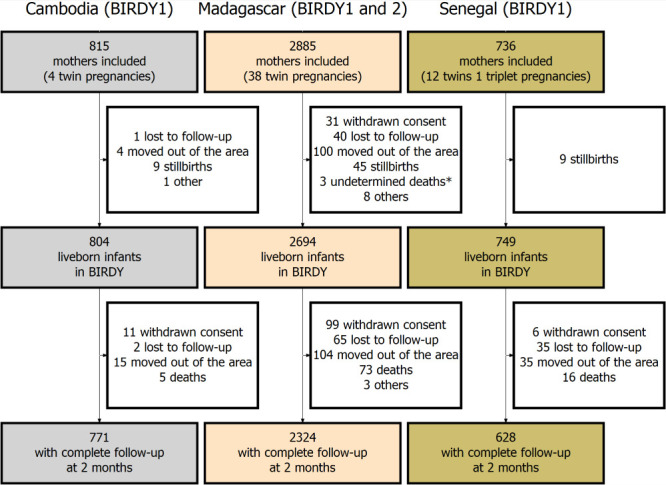
Flow-chart of combined BIRDY1 and BIRDY2 cohorts in Madagascar, Cambodia, and Senegal (2012-2021). *Three deaths of undetermined cause, that could not be classified as intrapartum stillbirth or early neonatal death.

We included 4436 mothers, 815 from Cambodia, 2885 from Madagascar, and 736 from Senegal (**Figure 2** and Supplementary Figure 1 in the **Online Supplementary Document**). At inclusion, 2.7% of mothers in Cambodia reported a history of stillbirth and 3.6% reported a history of death of at least one of their children, while these proportions were 4.5% and 7.4% in Madagascar, and 6.1% and 3.5% in Senegal (Supplementary Table 1 in the **Online Supplementary Document**). In Madagascar, deliveries outside health care facilities (41.8%) and/or without medical assistance (28.4%) were frequent, while they represented approximately 1% of cases in Cambodia and Senegal. The proportion of caesarean deliveries varied between countries, accounting for 12.4% of live births in Cambodia, 10.1% in Madagascar, and 3.4% in Senegal. Overall, 18% of pregnant women were recruited before 28WG and 67% before 37WG (Supplementary Figure 2 in the **Online Supplementary Document**).

In the BIRDY2 cohort (Madagascar), oligohydramnios was detected in 17 pregnant women (3.1%) and polyhydramnios in 18 (3.3%). Excluding antepartum stillbirth, 43 children (7.9%) had a breech presentation and 15 (2.4%) had a transverse presentation at the last ultrasound before pregnancy. The proportion of preterm birth was 16.1%.

### Incidence estimates and reported causes or circumstances

We presented the estimates of the incidence of stillbirth and neonatal death in **Table 1** and Supplementary Figure 3 in the **Online Supplementary Document**.

**Table 1:** Estimated incidence of stillbirth and neonatal death by country, with 95% confidence intervals

	Cambodia	Madagascar	Senegal
Early stillbirth (22WG-28 WG)*		11.4 (3.0-19.8)	
Stillbirth (>28 WG)*	11.0 (3.9-18.2)	10.4 (6.7-14.2)	11.9 (4.2-19.6)
Early neonatal death (0-7 d)†	5.0 (0.1-9.8)	21.9 (16.4-27.4)	12.0 (4.2-19.8)
Late neonatal death (8-28 d)†‡	-	3.6 (1.2-5.9)	8.3 (1.7-15.0)
Extended neonatal death (>28 d)†§	-	2.1 (0.3-3.9)	-
Neonatal deaths (0-28 d)†	6.2 (0.8-11.7)	25.2 (19.3-31.2)	20.0 (10.0-30.1)
All death (0-2 mo)†	6.2 (0.8-11.7)	27.1 (21.0-33.2)	21.4 (11.0-31.7)

WG – weeks of gestation, d – days, mo – months

*Per 1000 total births, BIRDY2 only.

†per 1000 livebirths.

‡One event in Cambodia.

§No event in Cambodia, one in Senegal.

#### Stillbirth

Among the 4436 pregnancies (including 55 multiple pregnancies), we documented 53 stillbirths. Three stillbirths in Madagascar were associated with maternal death (none in Cambodia or Senegal). Seven early stillbirths (22WG-28WG) were documented among the 613 mothers included in BIRDY2 before 28WG.

In Cambodia and Madagascar, 19 (43.2%) stillbirths were antepartum, 17 (38.6%) intrapartum, and eight (18.2%) undetermined (Supplementary Figure 4A in the **Online Supplementary Document**). Details of the circumstances were available for 24 stillbirths (42.1%). The most frequent circumstances were antepartum haemorrhage and complicated delivery (Supplementary Figure 4B in the **Online Supplementary Document**).

#### Neonatal mortality

**Figure 3:**
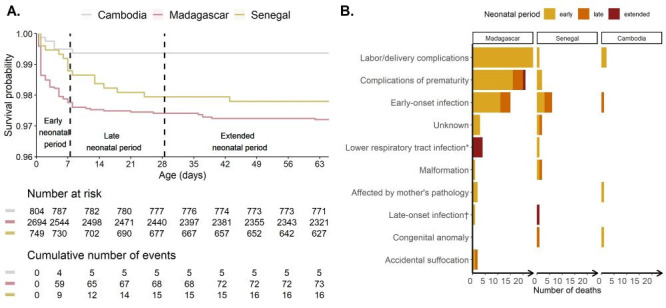
Neonatal mortality in Madagascar, Cambodia. and Senegal (BIRDY1/BIRDY2 cohorts, 2012-2021). **Panel A.** Survival curve of infants. **Panel B.** Possible causes of death. *Including bronchiolitis and pneumonia. †Including sepsis and meningitis.

Across countries, 94 of 4247 liveborn infants (2.2%) died before two months of age. Most deaths occurred during the early neonatal period (n = 7 9, 84%) and particularly during the first day of life (n = 46, 49%) (**Figure 3**, panel A). Sixteen additional deaths occurred during the late neonatal period (17%) and six occurred during the extended neonatal period (6%). While all deaths in Cambodia occurred in health facilities, 12.5% of deaths occurred outside health facilities in Senegal and 33.3% in Madagascar.

For early neonatal deaths, the main reported possible causes of death were complications related to labour or delivery (e.g., birth asphyxia) (34.2%), complications related to prematurity (25.3%), and neonatal infection (20.3%) (**Figure 3**, panel B). The main possible causes of death for late neonatal death were neonatal infection (based on symptoms, 50%) and complications related to prematurity (25%). For the few deaths that occurred during the extended neonatal period, reported possible causes of death were lower respiratory tract infection (bronchiolitis/pneumonia), meningitis, and complications related to prematurity. Of the deaths in BIRDY2, 61% occurred in premature infants.

### Associated risk factors

#### Stillbirth (>28WG)

**Figure 4:**
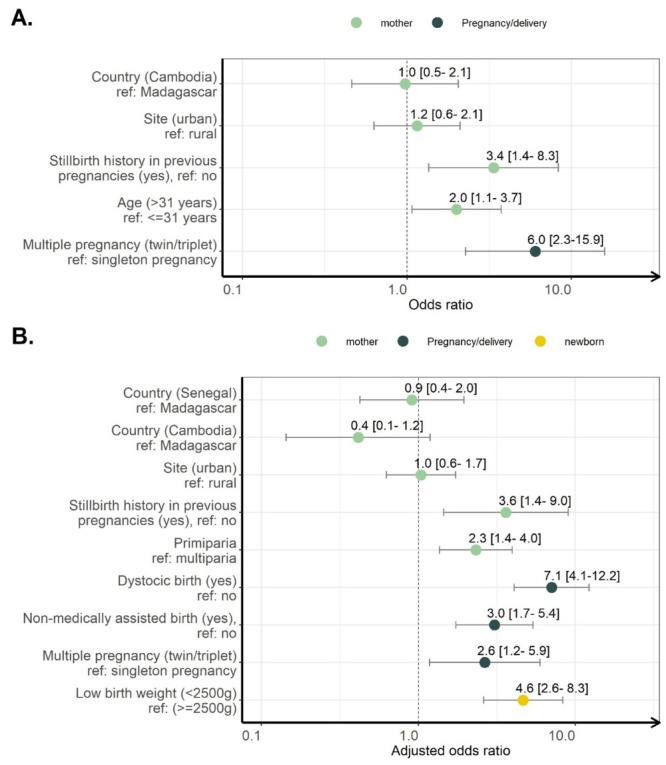
Risk factors associated with death from multivariate logistic regression (BIRDY1/BIRDY2 cohorts, 2012-2021). **Panel A.** Stillbirth (live birth = 3685, stillbirth = 44) in Madagascar and Cambodia. **Panel B.** Early neonatal death (survived = 4428, died = 72) in Madagascar, Senegal, and Cambodia (0-7 days).

Variables assessed as risk factors for stillbirth and results of univariate analyses are available in Supplementary Table 2 in the **Online Supplementary Document**. The variables associated with stillbirth in multivariate analysis were a history of stillbirth (aOR = 3.4; 95% CI = 1.4-8.3]), maternal age greater than 31 years (aOR = 2.0; 95% CI = 1.1-3.7), and multiple pregnancy (aOR = 6.0; 95% CI = 2.3-15.9) (**Figure 4**, panel A).

#### Early neonatal mortality (0-7 days)

Variables assessed as risk factors for early neonatal mortality and results of univariate analyses are available in Supplementary Table 3 in the **Online Supplementary Document**. The variables associated with early neonatal mortality were history of stillbirth (aOR = 3.6; 95% CI = 1.4-9.0), primipara mother (aOR = 2.3; 95% CI = 1.4-4.0), dystocic delivery (aOR = 7.1; 95% CI = 4.1-12.2), unassisted delivery (aOR = 3.0; 95% CI = 1.7-5.4), and low birth weight (aOR = 3.6; 95% CI = 2.6-8.3) (**Figure 4**, panel B).

## DISCUSSION

This prospective international cohort study is among the first to provide a comprehensive description of stillbirths and neonatal mortality in both community and health care settings, accounting for both rural and urban sites across a range of LMICs. The periods of delivery and the first week of life posed the greatest mortality risk, highlighting their importance for public health action. Documenting the burden of stillbirth and neonatal mortality is particularly challenging in settings where pregnant women lack access to care and in contexts of limited availability of medical resources. Our study design, which allowed for exhaustive community-based enrolment of neonates and their standardized longitudinal follow-up, has facilitated a more robust estimation of neonatal mortality than studies relying only on data from formal health facilities. This is particularly important for settings like Madagascar, where nearly 40% of women delivered outside of health facilities.

We estimated stillbirth incidences at 11 per 1000 total births in Cambodia, 15 per 1000 in Madagascar and 12 per 1000 in Senegal. Between 30% and 62% of stillbirths were intrapartum. These results are in line with data from recent population-based surveys, which have estimated the incidence of stillbirth to be 6 per 1000 in Cambodia (2014), 14 per 1000 in Madagascar (2008), and 18 per 1000 in Senegal (2018) [15-17]. Although estimated antepartum stillbirth rates in our study are high, they may nonetheless be underestimated: we included 33% of mothers after 37 WG, so antepartum stillbirths that occurred before potential enrolment were not documented. Earlier systematic recruitment may improve the accuracy of stillbirth incidence estimates (e.g., with preconception cohorts). This type of study has previously been implemented in Benin, with gestational age at recruitment of approximately 7 WG, but with a high rate of loss to follow-up that limits the reliability of the data [18].

We estimated neonatal mortality at 18 per 1000 live births in Cambodia, 24 per 1000 in Madagascar and 23 per 1000 in Senegal. These results are also in line with the most recently available population-based surveys (18 per 1000 in Cambodia, 25 per 1000 in Madagascar, and 21 per 1000 in Senegal) [2,6,12]. Comparatively low incidence in Cambodia reflects that this country has had one of the greatest declines in neonatal mortality recorded globally over the past two decades, reported as the result of improvements in the quality of prenatal care provided by trained health professionals, the introduction of tetanus toxoid injections during pregnancy, an increase in women's use of contraception resulting in an increase in the proportion of long-interval births, and a decrease in the proportion of women carrying children at risky ages [19].

The main reported causes of neonatal death were delivery complications (such as birth asphyxia), complications of prematurity, and neonatal infection. However, despite close follow-up of pregnant women in this study, causes of stillbirths remained mostly undetermined. Pathological examination of foetal and placental tissues is seldom performed in LMICs, making the identification of exact causes of stillbirth difficult [16]. In this context, the Child Health and Mortality Prevention Surveillance (CHAMPS) network is currently being implemented in seven LMICs which aims to systematically describe the causes of stillbirth and child death using minimally invasive autopsies with tissue sampling [20]. Although easier to implement than a complete autopsy, this method raises important logistical, ethical and cultural issues [21]. More data and the continued development of innovative strategies will be essential for better documentation of the causes of stillbirth and neonatal deaths in LMICs.

More than 90% of unattended deliveries in our cohort occurred in Madagascar, outside health facilities and in rural areas. We also observed that babies born without medical staff were three times more likely to die in the first week of life compared to babies delivered by a physician or a midwife. Delivering in a health facility allows for early and safer medical intervention during labour, especially in an emergency setting such as dystocic delivery. Moreover, training that targets medical staff and traditional birth attendants, including training on basic resuscitation practices, hygiene, and early detection of neonatal infection, could contribute to the reduction of perinatal mortality [22]. While geographic and financial barriers to accessing health facilities were minimized across all sites in our study, mothers’ choice of healthcare facilities was not explicitly influenced, and it is unclear why mothers in Madagascar sought formal care at much lower rates than mothers in Cambodia and Senegal. Anthropological studies could improve understanding of the factors guiding mothers to traditional healers so that effective interventions can be implemented to refer them to healthcare facilities, as has been done previously for the management of prematurity [23].

Schedules for antenatal visits were incomplete for most women, even though our study encouraged care-seeking during pregnancy and reduced barriers to care. It is estimated that only half of pregnant women worldwide complete the four antenatal visits previously recommended by WHO, and even fewer complete the eight visits that have been recommended since 2016 [7,22]. Besides various financial, organizational, and cultural barriers that are present in most LMICs, challenges in early pregnancy detection may contribute to insufficient follow-up. Improved development of family planning could allow earlier detection of pregnancy, leading to more prompt referral to appropriate health facilities, and could also prevent closely spaced pregnancies that are associated with higher risk of adverse birth outcomes [7,24].

We estimated gestational age by ultrasound in BIRDY2, which provided a reliable estimate of the gestational age at inclusion and prematurity rate among mothers in Madagascar. While the rate of premature birth has been estimated at around 11% in LMICs and 9% in HICs, it was 16% in this study, half of whom had low birthweight [25]. One ultrasound in early pregnancy (before 24 WG) is recommended by the WHO to identify multiple pregnancies, determine gestational age, and detect major foetal anomalies [26,27]. The implementation of ultrasound follow-up can allow for diagnosis and prevention of prematurity, but requires the acquisition and maintenance of equipment, the availability of personnel, and their training to perform this examination [26]. Considering the absence of early ultrasound examination and financial, geographical and cultural barriers to ultrasound access in many LMICs, accurately estimating the true incidence, causes, and consequences of prematurity is difficult.

### Strengths and limitations

This study has several limitations. Because gestational age was not available in BIRDY1, we did not assess risk of neonatal death associated with prematurity. We also did not weigh stillborn babies at birth because of the study design, precluding assessment of the proportion of those with low birthweight. Finally, due to insufficient sample size, we did not assess country-specific risk factors, and the risk factors identified in this work should be interpreted with caution as they may be country-specific.

Despite these limitations, our study has many strengths. Using standardized international definitions, we provided reliable estimates of stillbirth and neonatal mortality occurring in both healthcare and community settings. Through longitudinal follow-up initiated early in pregnancy in BIRDY2, we provided perhaps the first estimates of early stillbirth in Madagascar. Other study strengths are the inclusion of deliveries across all birth settings (hospital, primary health care centre or in the community) and across multiple countries and sites. Although we could not conduct autopsies relying on pathological findings, we systematically collected and reported circumstances of death by medical files and verbal autopsy, while paediatricians independently attributed the cause of death.

## CONCLUSIONS

In this international, multicentre cohort study, we estimated the incidences of stillbirth and neonatal mortality in three LMICs, accounting for neonatal deaths occurring in both community settings and in healthcare facilities. Appropriate interventions, such as the systematic monitoring of pregnant women and infants during the neonatal period, and the improvement of access to formal care facilities, could help reduce the overall incidence of stillbirth and neonatal mortality in these high-risk settings. To evaluate the effectiveness of such interventions, mortality surveillance must be reinforced to provide more accurate and better standardized estimates of the real number of deaths, including those occurring both within and outside of healthcare facilities.

## Additional material


Online Supplementary Document
