# Supplementary material for: Stillbirths and neonatal mortality in LMICs: A community-based mother-infant cohort study
Source: J Glob Health. 2023 Apr 14;13:04031. doi: 10.7189/jogh.13.04031 (PMC10103017; doi:10.7189/jogh.13.04031)
Supplement: Online Supplementary Document [file jogh-13-04031-s001.pdf]

Supplementary figures

Figure 1: Flow-chart of the BIRDY1 and BIRDY2 cohorts in Madagascar (2012-2021).

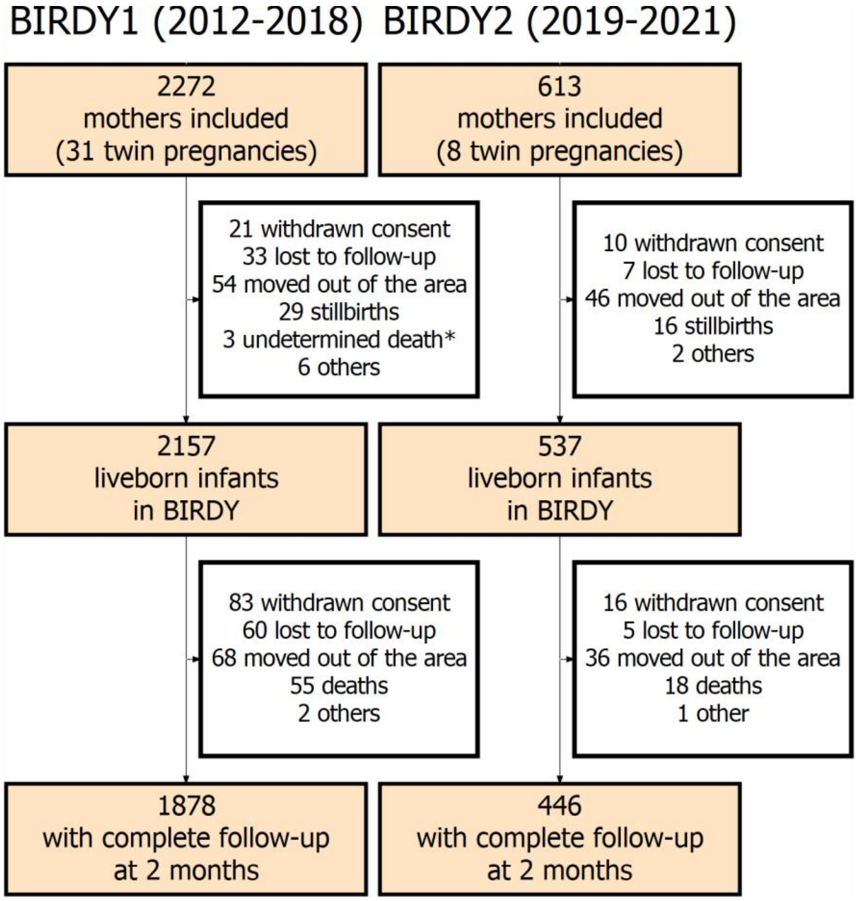

Figure 2: Timing of inclusion of pregnant women (n=4500 including 290 missing data).

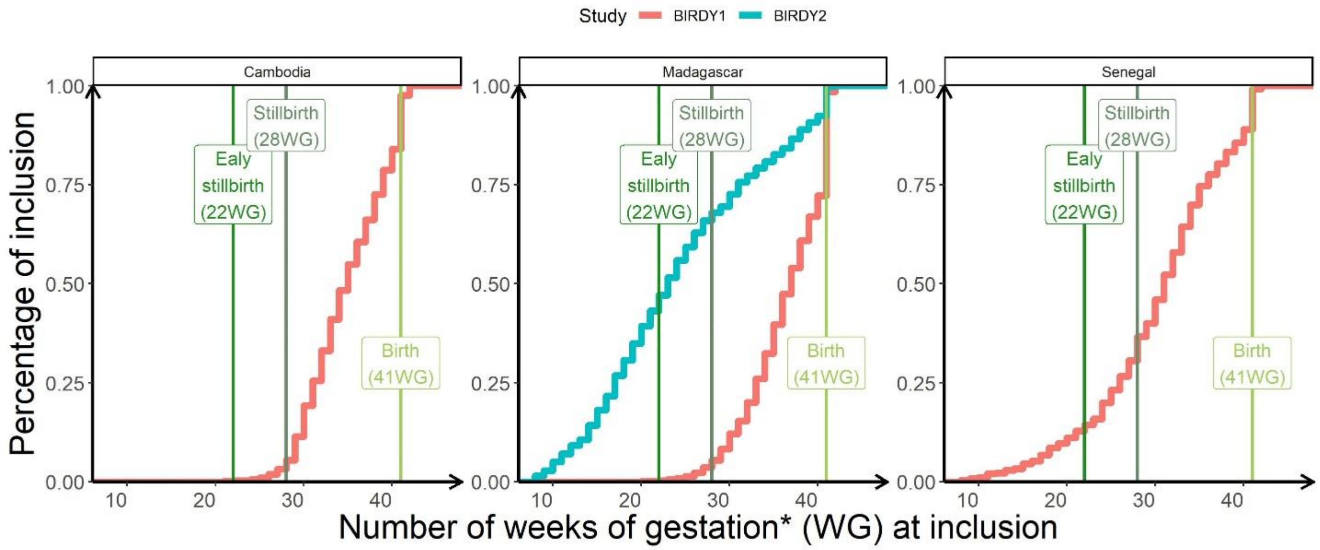

*Number of weeks of gestation assuming all pregnant women delivered at 41WG in BIRDY1 and according to first ultra-sound dating in BIRDY2.

Figure 3: Estimated incidence of stillbirths and neonatal death and confidence intervals in Senegal, Madagascar and Cambodia (BIRDY1-BIRDY2, 2012-2021) and in world bank geographic area (UN Inter-agency Group for Child Mortality Estimation, 2020).

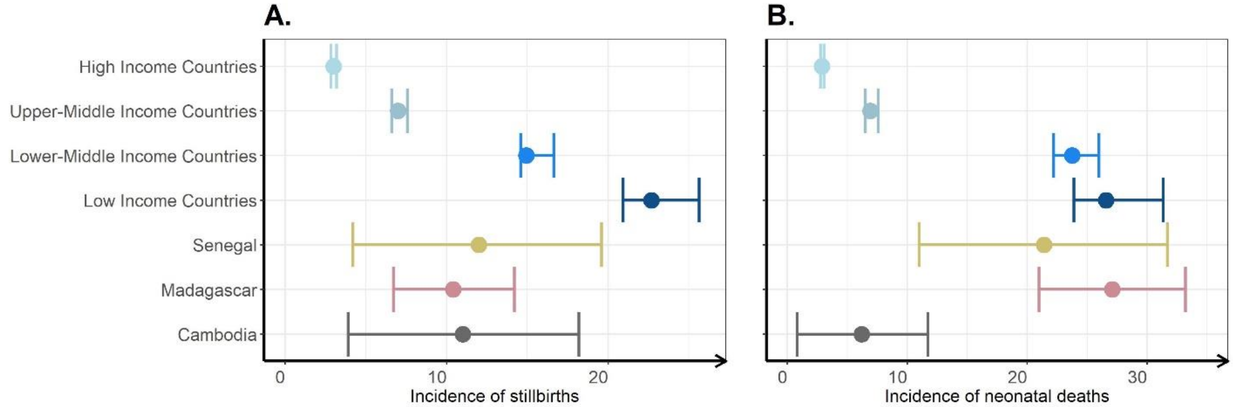

Data sources :
BIRDY1 and BIRD2 data, 2012-2021
UN Inter-agency Group for Child Mortality Estimation. Levels and Trends in Child Mortality. Report 2020. Published online 2020.

Figure 4: Stillbirths in Madagascar and Cambodia (BIRDY1/BIRDY2 cohorts 2012- 2021)

A. Percentage of stillbirth stratified by timing of fetal loss
B. Associated circumstances of stillbirth by timing of fetal loss

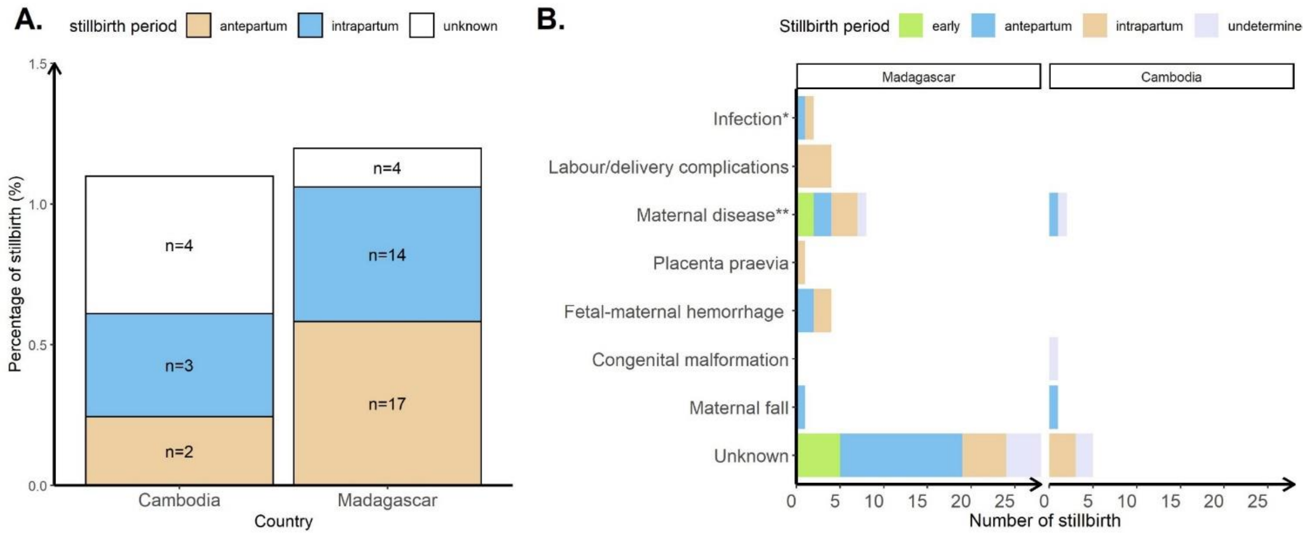

**Infection including Syphilis*
*** Maternal diseases such as hypertension*
sciwheel/placeholder/bibliography
y

Supplementary tables

Table 1: Socio-demographic characteristics at inclusion and at delivery by country (BIRDY1/BIRDY2 cohorts, 2012-2021) and biometric characteristics (BIRDY2 cohort only, 2019-2021)

Variable	Cambodia	Madagascar	Senegal	p	nNA[1]
**Mothers' characteristics**	**n=815**	**n=2885**	**n=736**		
Site (Urban)	370 (45.4)	1208 (41.9)	441 (60.0)	<0.001	9
Education level (<Primary)	370 (45.4)	2200 (76.3)	194 (26.4)	<0.001	9
Relationship Status (In couple)	795 (97.5)	2735 (94.8)	680 (92.6)	<0.001	11
Unemployed before pregnancy	279 (34.2)	1907 (66.1)	535 (72.8)	<0.001	10
Electricity at home (yes)	810 (99.5)	2193 (76.0)	713 (97.4)	<0.001	14
Latrine access (Outside)	405 (49.7)	2673 (92.7)	366 (50.1)	<0.001	16
Crowded house	193 (23.8)	461 (16.0)	38 (5.3)	<0.001	37
Smoker (first or second hand)	486 (59.6)	1316 (45.6)	178 (24.4)	<0.001	17
Age (mean (SD))	27.60 (5.45)	25.92 (6.47)	28.27 (6.48)	<0.001	10
Weight[2] (mean (SD))	49.89 (8.14)	52.22 (9.48)	63.72 (13.39)	<0.001	275
Primiparity	336 (41.2)	1080 (37.4)	191 (26.0)	<0.001	11
History of stillbirth	22 (2.7)	131 (4.5)	45 (6.1)	0.005	11
History of child death	29 (3.6)	214 (7.4)	26 (3.5)	<0.001	0
Number of visits during pregnancy (<4)	131 (17.0)	588 (20.7)	131 (18.1)	0.035	114
Iron supplements	782 (96.0)	2111 (73.3)	700 (95.8)	<0.001	17
Folate supplements	766 (94.2)	2012 (70.2)	496 (70.5)	<0.001	59
Multiple pregnancy (twins/triplets)	4 (0.5)	38 (1.3)	13 (1.8)	0.063	0
**Neonates' characteristics**	**n=804**	**n=2694**	**n=749**		
Antibiotic treatment per-partum	165 (22.4)	288 (11.5)	36 (5.7)	<0.001	365
Birth in healthcare facilities	789 (98.6)	1548 (58.2)	729 (98.4)	<0.001	40
Non-medically assisted birth	15 (1.9)	769 (28.6)	35 (4.7)	<0.001	12
C-section	99 (12.4)	271 (10.1)	25 (3.4)	<0.001	33
Foul-smelling amniotic fluid	16 (2.1)	199 (7.7)	115 (18.1)	<0.001	241
Dystocic Labor	36 (4.7)	242 (9.2)	14 (2.2)	<0.001	207
Sex (girl)	414 (52.1)	1307 (49.2)	368 (49.6)	0.363	54
Low birth weight (<2500g)	75 (9.4)	382 (14.4)	107 (14.6)	0.001	70
**Ultrasound data (BIRDY2 only)**		**n=620**			
Amniotic volume					67
Oligohydramnios		32 (5.7)			
Normal		503 (91.0)			
Polyhydramnios		18 (3.3)			
Fetal presentation[3]					76
Cephalic		478 (89.7)			
Breech		42 (7.9)			
Transverse		13 (2.4)			
Gestational age at birth[3] (<37WG[4])		98 (16.1)			78

1- *nNA = Number of missing data*
2- *Estimations of mother's weight before pregnancy*
3- *Excluding antepartum stillbirth (n^{total}=609)*
4- *WG : Weeks of gestation*

Table 2: Stillbirth (>28WG) risk factors in Madagascar and Cambodia (univariate and multivariate analysis)

	Livebirth	Stillbirths[1]	Crude OR CI95%	p-value	Adjusted OR CI95%	p-value
Number	3685	44				
Country (Cambodia)	810 (22.0)	9 (20.5)	0.9 [0.4-1.9]	0.808	1.0 [0.5- 2.1]	0.950
Site (urban)	1571 (42.6)	20 (45.5)	1.1 [0.6-2.0]	0.707	1.2 [0.6- 2.1]	0.642
Educational level (<Primary)	1122 (30.4)	19 (43.2)	1.7 [1.0-3.2]	0.072		
Relationship status (In couple)	3514 (95.4)	43 (97.7)	2.1 [0.3-15.2]	0.470		
Unemployed before pregnancy	2182 (59.2)	25 (56.8)	0.9 [0.5-1.7]	0.749		
Electricity at home (yes)	2986 (81.0)	36 (81.8)	1.0 [0.5-2.2]	0.932		
Latrine access (outside)	3065 (83.2)	37 (84.1)	1.1 [0.5-2.4]	0.872		
Crowded house[2]	660 (17.9)	7 (15.9)	0.9 [0.4-2.0]	0.732		
Smoker (first or second hand)	1803 (48.9)	17 (38.6)	0.7 [0.4-1.2]	0.178		
Age (>31 years)[3]	865 (23.5)	18 (40.9)	2.3 [1.2-4.1]	0.009	2.0 [1.1- 3.7]	0.029
Weight before pregnancy (50kg)[4]	1708 (46.4)	17 (38.6)	0.7 [0.4-1.3]	0.227		
Primiparity	1415 (38.4)	15 (34.1)	0.8 [0.4-1.6]	0.560		
Stillbirth history	145 (3.9)	6 (13.6)	3.9 [1.6-9.3]	0.003	3.4 [1.4- 8.3]	0.009
Child death history	235 (6.4)	7 (15.9)	2.8 [1.2-6.3]	0.015		
Number of prenatal consultations (<4)	736 (20.0)	10 (22.7)	1.0 [0.5-2.2]	0.985		
Iron supplement	2880 (78.2)	36 (81.8)	1.3 [0.6-2.7]	0.565		
Folate supplement	2781 (75.5)	32 (72.7)	0.9 [0.4-1.7]	0.666		
Multiple pregnancy	78 (2.1)	5 (11.4)	5.9 [2.3-15.4]	<0.001	6.0 [2.3-15.9]	<0.001

1- *Stillbirth (>28WG)*
2- *More than 4 persons by bedroom*
OR *CI95% = Odd ratio, Confidence interval at 95%*
3 - >3rd quartile
4 -> 1st quartile

Table 3: Early neonatal mortality (0-7 days) risk factors in Madagascar, Cambodia and Senegal (univariate and multivariate analysis)

	Alive at day 8	Early neonatal death	Crude OR CI95%	p-value	Adjusted OR CI95%	p-value
Number	4175	72				
Country (Cambodia)	740 (17.7)	9 (12.5)	0.2 [0.1-0.6]	0.004	0.4 [0.1- 1.2]	0.101
(Senegal)	2635 (63.1)	59 (81.9)	0.5 [0.3-1.1]	0.090	0.9 [0.4- 2.0]	0.809
Site (urban)	1892 (45.3)	30 (41.7)	0.9 [0.5-1.4]	0.538	1.0 [0.6- 1.7]	0. 879
Maternal educational level (<Primary)	1589 (38.1)	25 (34.7)	0.9 [0.5-1.4]	0.563		
Mother unemployed before pregnancy	2557 (61.2)	55 (76.4)	2.0 [1.2-3.5]	0.010		
Electricity at home (yes)	3520 (84.3)	51 (70.8)	0.5 [0.3-0.8]	0.003		
Crowded house[2]	664 (15.9)	14 (19.4)	1.3 [0.7-2.3]	0.392		
Smoker (first or second hand)	1865 (44.7)	27 (37.5)	0.7 [0.5-1.2]	0.225		
Mother's age <1st quartile (21 years)	890 (21.3)	26 (36.1)	2.1 [1.3-3.4]	0.003		
Primiparity	1478 (35.4)	41 (56.9)	2.4 [1.5-3.9]	<0.001	2.3 [1.4- 4.0]	0.002
Stillbirth history	173 (4.1)	7 (9.7)	2.5 [1.1-5.5]	0.024	3.6 [1.4- 9.0]	0.006
Child death history	244 (5.8)	5 (6.9)	1.2 [0.5-3.0]	0.694		
Multiple pregnancy (twins/triplets)	95 (2.3)	10 (13.9)	6.9 [3.4-13.9]	<0.001	2.6 [1.2- 5.9]	0.018
Sex (boy)	2096 (50.2)	41 (56.9)	1.4 [0.8-2.4]	0.203		
Non-medically assisted birth	796 (19.1)	28 (38.9)	2.6 [1.6-4.4]	<0.001	3.0 [1.7- 5.4]	<0.001
Birth in healthcare facilities	3032 (72.6)	47 (65.3)	0.7 [0.4-1.2]	0.242		
Antibiotic pre-partum	530 (12.7)	10 (13.9)	1.3 [0.7-2.6]	0.424		
C-section	388 (9.3)	9 (12.5)	1.5 [0.7-3.0]	0.272		
Foul smelling amniotic fluid	336 (8.0)	9 (12.5)	1.6 [0.8-3.3]	0.216		
Dystocic birth	279 (6.7)	26 (36.1)	8.4 [5.1-13.9]	<0.001	7.1 [4.1-12.2]	<0.001
Low birth weight (<2500g)	547 (13.1)	33 (45.8)	6.1 [3.6-10.3]	<0.001	4.6 [2.6- 8.3]	<0.001

1- *More than 4 people per bedroom*

OR *CI95% = Odds ratio, Confidence interval at 95%*

Table 4: Late and extended neonatal mortality (8 days – 2 months) risk factors in Madagascar and Senegal (univariate and multivariate analysis)

	Alive at 2 months	Extended/late neonatal death	Crude OR CI95%	p-value	Adjusted OR CI95%	p-value
Number	3225	21				
Country (Senegal)	714 (22.1)	7 (33.3)	1.8 [0.7- 4.4]	0.225	1.9 [0.7- 5.0]	0.209
Site (urban)	1455 (45.1)	11 (52.4)	1.3 [0.6- 3.2]	0.506	1.0 [0.4- 2.4]	0.940
Maternal educational level (<Primary)	1110 (34.4)	8 (38.1)	1.2 [0.5- 2.8]	0.724		
Mother unemployed before pregnancy	2180 (67.6)	10 (47.6)	0.4 [0.2- 1.0]	0.058		
Electricity at home (yes)	2600 (80.6)	20 (95.2)	4.8 [0.6-35.9]	0.126		
Crowded house[2]	453 (14.0)	5 (23.8)	1.9 [0.7- 5.3]	0.206		
Smoker (first or second hand)	1329 (41.2)	7 (33.3)	0.6 [0.2- 1.7]	0.350		
Mother's age <1st quartile (21 years)	746 (23.1)	7 (33.3)	1.7 [0.7- 4.1]	0.275		
Primiparity	1079 (33.5)	7 (33.3)	1.0 [0.4- 2.5]	0.999		
Child death history	209 (6.5)	2 (9.5)	-	-		
Multiple pregnancy (twins/triplets)	82 (2.5)	6 (28.6)	15.3 [5.8-40.5]	<0.001		
Sex (boy)	1637 (50.8)	7 (33.3)	0.5 [0.2- 1.2]	0.120		
Birth unassisted by medical staff	732 (22.7)	4 (19.0)	0.8 [0.3- 2.4]	0.691		
Birth in healthcare facilities	2158 (66.9)	15 (71.4)	1.2 [0.5- 3.2]	0.666		
C-section	282 (8.7)	0 (0.0)	-	-		
Foul smelling amniotic fluid	305 (9.5)	3 (14.3)	1.6 [0.5- 5.5]	0.454		
Dystocic birth	233 (7.2)	2 (9.5)	-	-		
Low birth weight (<2500g)	432 (13.4)	13 (61.9)	11.2 [4.5-27.9]	<0.001	8.5 [3.3-21.9]	<0.001
Weight loss >10% at day 3	406 (12.6)	7 (33.3)	4.1 [1.6-10.8]	0.004	4.4 [1.6-12.1]	0.005
Exclusive breastfeeding up to day 7**	2924 (90.7)	13 (61.9)	0.1 [0.1- 0.4]	<0.001		
**Event between birth and day 7**						
Respiratory distress	140 (4.3)	5 (23.8)	6.9 [2.5-19.1]	<0.001		
Fever	269 (8.3)	8 (38.1)	6.8 [2.8-16.5]	<0.001		
Antibiotic treatment	417 (12.9)	10 (47.6)	6.1 [2.6-14.5]	<0.001	5.7 [2.3-14.3]	<0.001
Icterus	179 (5.6)	6 (28.6)	6.8 [2.6-17.8]	<0.001		
Hospitalization	111 (3.4)	6 (28.6)	11.2 [4.3-29.5]	<0.001	8.0 [2.7-23.4]	<0.001

1- *More than 4 persons per bedroom*

OR *CI95% = Odds ratio, Confidence interval at 95%*
